# Artificial Intelligence, Brains, and Beyond: Imperial College London Neurotechnology Symposium, 2020

**DOI:** 10.1089/bioe.2020.0040

**Published:** 2020-09-16

**Authors:** Anna Tarasenko, Mikheil Oganesyan, Daryna Ivaskevych, Sergii Tukaiev, Dauren Toleukhanov, Nickolai Vysokov

**Affiliations:** ^1^ BrainPatch Ltd., London, United Kingdom.; ^2^ Research Institute, National University of Ukraine on Physical Education and Sports, Kyiv, Ukraine.

**Keywords:** neurotechnology, artificial intelligence, brain-computer interface

## Abstract

In this report, we give an overview of the proceedings from the online Imperial College London Neurotechnology Symposium 2020. The first part deals with the fundamentals of how artificial intelligence (AI) can be used to inform research frameworks used in the field of neurotechnology. The second part goes a level higher and shows how AI can be used in cutting-edge cellular and molecular methodologies and their applications. The final part focuses on the efforts to “decode” neural systems in brain-computer interfaces to advance neuroprosthetics.

## Introduction

Every year Imperial College London invites some of the brightest minds from around the world to share their exciting discoveries and updates at the Neurotechnology Annual Research Symposium organized by the Centre of Excellence in Neurotechnology. The 2020 event, however, was slightly different. Owing to the ongoing pandemic it was organized online, but still with attendees from across the globe. The backgrounds of the invited speakers ranged from molecular and cellular neurobiology to industrial consumer-oriented product developers. The quality of the talks was outstanding.

In this report, we give an overview of the proceedings. The first part deals with the fundamentals of how artificial intelligence (AI) can be used to inform research frameworks used in the field of neurotechnology. The second part goes a level higher and shows how AI can be used in cutting-edge cellular and molecular methodologies and their applications. The final part focuses on the efforts to “decode” neural systems to advance neuroprosthetics.

## AI and Its Potential Impact in Neurotechnology

As an umbrella term, AI covers a wide range of topics. A common convention is to include techniques such as statistical learning and traditional learning algorithms (e.g., support vector machine) into the machine learning (ML) subset. Newer techniques such as “deep learning” and “reinforcement learning” are often considered to be separate. The common aim, however, is ultimately to solve complex problems, matching or even surpassing human performance.^1^

The opening talk was given by Dr. Romy Lorenz (University of Cambridge, Stanford University and the Max Planck Institute for Human Cognitive and Brain Sciences). She presented a novel technique in neuroadaptive technology using Bayesian optimization for the expansion of experimental space. The technique falls under the umbrella term of AI and grants a more holistic understanding of human cognition as it prevents overspecified brain functionalization.^2^

The speaker identified three potential areas of application of this approach: improvement in human brain mapping, development of reliable biomarkers of particular brain states, and creating a more individual approach toward noninvasive brain stimulation. When it comes to brain mapping, one should first decide on the brain state to optimize for. For example, if one wants to achieve maximum activation of a particular area of visual cortex, then the real-time data analysis is applied to the functional magnetic resonance imaging data and a search through the space of different conditions (e.g., transcranial alternating current stimulation frequency and phase pairs) is conducted to find the condition leading to the highest activation in the chosen area of the brain. Traditionally, the “map” of responses showing the most and least effective conditions would be calculated through exhaustive grid search or random search approaches. Instead, the author suggested using the process of search optimized through Bayesian optimization to find the most effective conditions in the least number of steps.

This algorithm (called the “surrogate function”) tries to approximate the brain map by sampling various states (e.g., stimulus-response pairs) one step at a time. The more samples are drawn, the more accurate the surrogate function becomes. However, unlike grid and random search, Bayesian optimization uses an acquisition function to control where in the sample space the next sample will be drawn. The latter is chosen in the area of the highest uncertainty, that is, where the expected improvement to the surrogate function is the highest. This way, it is possible to converge to the optimum condition in the least number of steps.

One of the assumptions made is that similar experimental conditions will elicit similar brain states. This approach allows isolation of individual networks involved in particular tasks. This talk was concluded with more general implications of the technique and a framework for conducting experiments to help avoid some of the ill practices in the field, for example, SHARKing (“Selecting Hypothesized Areas after Results are known”).

Many different laboratories have been utilizing some form of AI/ML. However, the fundamental problem is that the newly popular deep learning algorithms are a black box and do not provide any insights into the decision making. A noteworthy poster presentation by Tamara Gerbert (from the laboratory of Prof. Anil Anthony Bharath, Imperial) dealt with the comprehension of deep learning models,^3^ a topic that has been steadily gaining attention at the highest level in the research community. More specifically, the author focused on deep reinforcement networks (also known as “deep Q-learning reinforcement networks” or DQNs) with a proof-of-concept example centered around a robotic arm interacting with a ball in a virtual environment.

DQNs combine standard reinforcement learning architectures based around action-reward policies with conventional dense and/or convolutional layers that have an inherent advantage of automatically learning complex feature representations. Unfortunately, it is often difficult to humanly understand what the learned representations are! To demystify the contents of the hidden layers, the author monitored them under various conditions within the virtual environment. For example, by perturbing the color of the virtual ball and observing the points of interest, it was possible to judge the significance of the object's color in the decision-making process of the DQN. The ability to have such quantifiable insight cannot be overstated as the heuristics can be used to repair the shortcomings of the models by either architectural changes or domain adaptation.

## Artificial Brains, Model Organisms, and Their Impact on Fundamental Research

Despite its complexity, human brain shares distinct features with brains of simpler organisms, which, therefore, can be used as models to understand fundamental processes. One of the simplest models is the worm, *C. elegans*. This species multiplies rapidly and its behavior can be studied live under the microscope, including with high-throughput screening assays. To automate the image analysis, Ziwei Li (co-supervised by Prof. Anil Bharath and Dr. Andre Brown) presented a poster reporting the use of Deep (8-layer) Convolutional Neural Networks to tell if a certain number of pixels belongs to the image of the worm whereupon its movements could be tracked.^4^ This method could ultimately be used to study worm behavior and screen drugs in 96-well plates.

Mice are another popular model that can be used to study the neural circuitry in development. Moreover, with modern optogenetic tools it is possible to visualize the activity of neurons and to stimulate them. One challenge, however, lies in closed-loop recording and stimulation. Isabell Whiteley (Dr. Chris Rowlands' laboratory) presented a system of lasers and digital micromirror devices to project holograms at multiple depths simultaneously. She showed that it is thus possible to record electrical activity in dendrites. This technology, when applied to freely behaving animals, can lead to an unprecedented level of control over brain activity.

The biggest criticism of animal models is that the findings do not always translate accurately to humans especially in a clinical setting. Madeline Lancaster is the original developer of human brain organoids.^5^ In her talk, she presented updates on the efforts of her laboratory to improve the technique to increase reproducibility and has described some of its novel features and applications. Specifically, these organoids were able to connect to muscle tissues and send signals to the muscles to make them twitch. A particularly exciting part was the discovery of cells within these organoids that resembled the choroid plexus. The latter serves to produce cerebrospinal fluid. Interestingly, also, the organoids contained the receptor for the SARS-CoV2 virus. Thus, the organoid technology can be used to gain insights to neurological effects of infection and to develop drugs facilitated by high-throughput screening.

## Cutting Edge Research on Brain–Computer Interfaces

Brain–computer interfaces (BCIs) represent a technology built on the idea that brain-generated signals can be interpreted and used to produce a desired behavior of a machine. This is particularly relevant for people with compromised neural output pathways by allowing creation of neuroprosthetics. Despite all the advances that appeared in this field, however, lack of fundamental understanding of the behavior-encoding mechanisms could be limiting. Dr. Juan Alvaro Gallego (Bioengineering, Imperial) addressed this problem from a perspective of neural populations. He presented a revolutionary view on studying the control of movement.^6^

First to highlight were the challenges faced by the neuroprosthetic research, in particular the problem of neural turnover. Although an array of electrodes could be inserted in the motor cortex and calibrated to pick up and interpret signals that code for movement, the accuracy of the decoder declined with time. This problem seemed to arise because single-neuron coding was used and this represented different features of movement, such as speed and direction. The authors proposed an alternative theory that the movement planning and execution was carried out through patterns of neuronal cluster activity referred to as “neural modes.”^7^

The trajectories in which activity of the neural modes can develop then forms a neural “manifold.” If the modes that span the manifold can be identified, these can carry out the stable decoding of the intended movement. Consequently, any migration of neurons that happens often in the course of recordings would no longer matter. Application of this technique to the motor cortex of monkeys gave promising results. This research could be particularly important for neuroprosthetics and their use on people with locked-in syndrome. The talk was concluded with a wider discussion of applications to the process of motor learning. It was hypothesized that the long-term learning of a new action involved formation of new neural modes. These would be harder than short-term adaptation needed to change an already familiar movement using existing neural modes.

The talk by Patrick Kaifosh started with the brief history of the company called CTRL-Labs and their vision to understand and address the discrepancy between the information that humans can sense and output. To decrease the information loss and output latency, researchers directly analyzed the signals sent to the motor units using electromyography (EMG). Action potentials conducted at the innervation zone of the motor unit could be sensed through the skin of the muscle using dry electrodes. The detected signals were used to decode and track wrist and finger movements, accurately estimating their speed and force. These were then used to create interfaces enabling subtle finger movements to operate a computer interface.

CTRL-Labs merged with the Facebook Reality Labs to create a more complete experience of Virtual Reality. Recently, the laboratory has been working to further improve detection of the EMG signals and make the system robust to potential movement of the wearable on the wrist. An important question was how this approach differed from simply measuring muscle tension!

Classical size principle of the recruitment of the motor units would state that the larger motor units are recruited last as they have a higher threshold of activation. It means that their voluntary control is not readily possible. Researchers have shown, however, that when there is another source of feedback about the motor unit activity, it is possible to train an individual to control the activity of the multiple motor neurons within a single muscle individually. Conceptually, this means that it might be possible to augment human output through enabling new ways of controlled information output.

When studying neural dynamics, we not only want to be able to just understand it but also have the means to influence it. An example of such a technique is “deep brain stimulation” (DBS). This is widely used in clinical practice due to its high-level efficiency.^8^ However, the invasive nature of DBS limits its accessibility to a small number of medical institutions where neurosurgeons can collaborate with psychiatrists.^9^ The development of noninvasive technologies with a simpler procedure is necessary, therefore, to make the treatment of severe psychiatric conditions more affordable for patients.

Patrycja Dzialecka's study of temporal interference (TI) of electric fields is dedicated to the functioning of this noninvasive brain stimulation method and neuronal processes that confer its effects. She presented her findings on the biophysics of neural stimulation through TI of electric fields, which is the result of the superposition of electric fields with frequencies differing by not >1 kHz. Low-pass filtering of the signals within neurons would prevent high-frequency oscillations in neural tissues.^10^ Current research is aiming at investigating the mechanisms of TI that can be realized by a linear or nonlinear combination of inputs (or both) and measuring strength and focality of TI stimulation *in vivo*. According to the model of spiking neurons developed, the interconnectivity of the neurons may result in a small increase in the response to the frequency difference and introduce further harmonics.

## Concluding Remarks

A common theme running through most of the talks was the use of AI algorithms to solve a variety of tasks that were previously done manually or were thought to be impossible. This has value for fundamental and applied neurotechnology. Although there are many attempts to drive such technology products to the mass market, to the best of our knowledge none has got there yet. This could change, however, as early as 2021. One of the candidates to do so in the foreseeable future is probably CTRL-Labs, a neurotechnology company recently acquired by Facebook for an undisclosed amount believed to be $500M–1B. Facebook is known to think strategically and neurotechnology seems firmly on its agenda. It is likely that other IT corporations will follow suit and start looking into fundamental and applied neurotechnologies, including Bayesian optimization for BCIs or whole-brain imaging and stimulation, and even artificial brains.

All these could spell the beginning of a new era in neuroscience and its business. At BrainPatch, we believe that using AI is critical for developing BCIs. To lead the field, however, new meta-technologies must emerge by unifying the efforts of various neuro companies and researchers, thus unravelling the true potential of neurotechnology.
